# Crystalising employment quality and behavioural outcomes of employees in the public service

**DOI:** 10.1016/j.heliyon.2020.e05619

**Published:** 2020-12-19

**Authors:** O.P. Salau, A. Osibanjo, A. Adeniji, H. Falola, E. Igbinoba, T. Atolagbe, O. Ogueyungbo

**Affiliations:** Business Management Department, Covenant University, Ota, Ogun State, Nigeria

**Keywords:** Employment quality, Culture, Rewards, Relationships, Employee behavioural outcome, Policy, Social sciences, Economics, Business, Psychology, Sociology

## Abstract

The goals of equitable and fair treatment in employment relationships have become a growing concern due to demographic changes and globalisation. Consequently, the quality of employment and relationships appeared to be an anachronism in competitive conditions where an organisation may need to minimise costs by reducing the headcounts. The study investigated the determinants of employment quality on employee behavioural outcomes. The study adopted a descriptive research design to establish trends related to the objectives of this study. The survey questionnaire was adopted to elicit information from the staff of the selected public (corporation) service. Out of 350 copies of the questionnaire distributed, only 230 copies were duly filled and completed. A variance-based model, specifically, SEM was adopted to establish goodness fit. Results indicate that work flexibility, learning and development, organisational culture, involvement in the determination of reward system, and work relationship significantly impact on employee behavioural outcomes. On the contrary, it was evident that the organisational policy has the least predictive value for promoting desirable outcomes. This could probably be as a result of many written rules and bureaucratic structure that exist in the public service. Differences in employees’ behavioural outcomes can be linked to age (λ^2^ = 5.741, df = 4, P < 0.05), educational background (λ^2^ = 7.723, df = 4, P < 0.05) and work experience (λ^2^ = 9.672, df = 4, P < 0.05) while gender was not statistically significant. By implications, organisational policies should be tailored towards ensuring the quality of employment and should be targeted to facilitate commitment and positive work behaviour.

## 1 Introduction

With the growth of competitive and free-market conditions imposed on developing countries, their enterprise managers and government are now being compelled to appreciate the crucial importance of rational goal setting that is based on the applications of the principles of external and external environment scanning. There tends to be a high-speed and swift rate of globalisation and innovation in a business environment, and these have tremendously increased the level of competition in the organisation. There is a total shift from the traditional business environment, which we all know, to a more competitive one, which involves the internal environment majorly. Human resources are regarded as an essential internal resource to an organisation (Lawrence and Lorsch, 1967; Danish and Usman, 2010; Osibanjo et al., 2016; Salau et al., 2020). The human capital of any country represents an essential asset for the organisation and are the most incredible resource for achieving organisational growth and sustainable development goals (SDGs). This idea was emphasised by Salau (2017), who believed that a country most crucial treasure is its human resources. This gives a full explanation on why developed nations pay utmost attention to human resources than other factors such as land, capital and entrepreneur. This clearly shows that people are vital to means of production, and they, therefore, merit equitable treatment, respect and adequate welfare schemes and inducements (Salau, 2017; Adeniji et al., 2018).

Organisations that aspire to achieve sustainable performance are required to provide dependable answers to this salient question: What best can we do to motivate exiting and intending employees to keep longing for our organisation? Most employees are driven by how organisations value them than the pay they receive (Akinade and Tunde, 2011; Daneshfard and Ekvaniyan, 2012; Sanjeev and Surya, 2016; Samwel, 2018). Therefore, the effective and efficient allocation of resources contributes to a significant positive effect on the employment relationship (Abdali, 2011; Ibidunni et al., 2016). As a result of the competitive dynamism found in the present business world of today, the effective management of limited skills, competences, the desirability and retention of trained employees, is the most significant issues in human resource management. The organisation derives pride in the satisfaction of its employees, as it is a critical factor in ensuring they get the best out of these workers, in the processes of achieving its goals and objectives. Inevitably, specific procedures and policies need to be directed towards employment quality and relationships. This has become an important means of ensuring that people are effectively utilised to achieve individual or personal and group or corporate objectives (Adeniji and Osibanjo, 2012; Ahmad, 2013; Akinade and Tunde, 2011). One of the fundamental worries of human resource management (HRM) and the utmost prominence is the quality of employment.

Employment quality can be explained from two folds: The first focuses on the concept of "job quality or characteristics (i.e. job contents, skill taxonomy, autonomy, job contexts, resources and expectations) (Akinade and Tunde, 2011; Sanjeev and Surya, 2016; Osibanjo et al., 2020); while the second is associated with the terms of employment and relations (e.g. salary administration, pension rights, contract, working hours, relationship, trust). This implies that amplified stress on flexibility and adaptation to the changing nature of employment in recent decades has compelled the need for organisations to assess the consequences of "job quality and employment situations (Lunenberg, 2011; Madu and Dike, 2012; Rainey, 2014). Where there is employment quality, individuals feel connected to the organisation and are committed to its goals. Providing employment quality may appear to be an anachronism in competitive conditions where every organisation and/or sector may need to reduce costs by reducing the headcounts. Studies argued that it is vital for a number of reasons (Ibrahim et al., 2013; Abdulla et al., 2010). Although the Nigerian public service has experienced transformational changes over the years, surprisingly successive restructurings have not had any significant influence on reengineering the public sector.

In public parastatals, where process discipline is classically inadequate, the process of managing people for increased performance will still become a futile activity. That is not to argue that there is no place in the Nigerian public sector for so-called modern management practices. As the HR function is one means of facilitating employment quality and gaining competitive advantage, two questions arise: Why should there be particular interest in HRM in the Nigerian Public service? Why not apply the principles and practices of HRM in general terms to both corporate and public institutions? Yes, there are many HRM principles and practices that are generally applicable. However, some are not due to the very nature of public service. Its achievement is different from those of a corporate organisation. Its capacity to experiment, innovate and move with the time is circumscribed. Reforms and changes occur infrequently. Employees regard government business as nobody's business, and quite often, their survival (retention on the job) may not directly be dependent on individual performance or government financial viability.

Importantly, understanding the best approach to adopt in attracting, developing and retaining knowledgeable talent have posed a more significant challenge to employment quality and relationships. Review from extant literature showed that dearth research had been carried out on how quality employment can be used to facilitate desired employee behavioural outcomes in the public sector. However, dearth studies conducted in the field of job quality and performance has covered domains such as telecommunications, education, and banking. Still, not much has been carried out in the government parastatal (public sector). It is against this background that this study examined the combined effect of employment quality dimensions (work flexibility, organisational culture, learning and development, involvement and rewards, work systems, work relationship) on employee's behavioural outcomes in the public sector. To achieve this, the following research questions were formulated to guide the study:

i. To what extent can work flexibility and organisational culture influence on employee behavioural outcomes?
ii. In what ways can learning and development and organisational policy affect employee behavioural outcomes?
iii. To what extent can equitable rewards and work relationship influence employee behavioural outcomes?
iv. What are the employees' perceptions of employment quality and employee behavioural outcomes across the demographic characteristics?

## 2 Literature review

### 2.1 Employment quality defined

Employment quality is a difficult concept which is multi-dimensional. Their definition and components depend on the perspective taken, i.e. whether employment quality is evaluated based on the view of society, business or individuals. The social perspective of good jobs focuses on the social spin-offs of good or bad jobs. "Good" quality jobs for society may mean employment which is sufficient for the skills of the workers, resulting in high rates of productivity and improving social cohesion (Aluko, 2007; Long et al., 2012; Kaufman et al., 2013; Jerrell et al., 2014). In company terms, a "good" quality job can be matched by a qualified and efficient workforce. The quality of their employment can be rated high by individual workers if certain conditions such as safety and health are met. There is an overlap between the views of the company and the worker about quality jobs. For example, both dimensions of job quality are income and benefits from employment, as well as job security and social protection (Rani and Mishra, 2012; Salau et al., 2014; Salau et al., 2018).

To put merely, employment quality can be defined as a multi-dimensional phenomenon used to determine at different institutional levels factors that can affect the well-being and relationships between the employers and employees. Employers and employees have different criteria for determining the standard of jobs, based on social and economic conditions, group memberships and individual preferences. For society, policymakers, Governments and researchers, quality of jobs is an important issue. The heterogeneity of jobs has increased in many countries during recent decades. Non-standard employment types have significantly increased, while the proportion of standard employment with contracts have fallen. These questions emphasise the importance of extra systematic information on job quality to supplement the well-established quantitative labour market indicators. Employment is not just in terms of time and income but is central to people's lives in many countries (Shakeel and But, 2015; Adeniji et al., 2020). In many respects, employment impact significantly on the quality of life. Often it is vital to social and financial progress; it offers people identity and social opportunities. Jobs are not risk-free at the same time. The employment situation of different demographic and social population groups in many countries differs significantly.

Many international organisations emphasise the importance of job quality. Promoting decent work is the primary purpose of establishing the International Labour Organization (ILO). The main reason for establishing ILO is to acquire decent work and encourage equal opportunities for both men and women. This statement makes it quite evident that the work of the International Labour Organization goes far beyond the concern for the number of jobs and focalises on workers’ rights, social protection and social dialogue. Generally, employment quality can be seen as a strategic approach focused on skills development, equal compensation, development opportunities, learning & progression, health and safety security, flexibility in the workplace, autonomy & control, culture of respect and the security of employment (Asmui et al., 2012; Knudsen et al., 2011; Nahavandí, Denhardt, Denhardt and Aristigueta, 2015).

To some extent, the quality of employment is subjective and idiosyncratic and reflects the fit between the needs of specific workers and the characteristics of specific jobs. Since individuals spend a large fraction of their lifetime on the job. The quality of the job thus is one of the most critical determinants of the quality of life, as presented in Table 1.

**Table 1:** Dimensions of quality of employment framework.

Dimensions of Quality of Employment Framework	Description	Example
Competitive Compensati0n & benefits Attractive	….compensation plays a significant role in retaining top talent in an organisation. If what the firm offer is not competitive enough, they might be forced to leave. Employees should be rewarded based on what they want, in the form of pay, perks and many more and not what the organisation feel they should give them (Employee Value Proposition).	▪ Compensation should be based on merit and not favouritism. It should be based on employee's skills, competencies, experience, qualifications, etc. ▪ Flexibility is a must in any compensation plan. It should be flexible in a way that allows for change as environmental variables changes from time to time. ▪ Machineries for resolving compensation issues must be precise and well communicated to all
Promoti0nal opportunities and career progression	Succession planning develops employee's career, and this is the more reason why employees are interested in and the positions they might get in future.	▪ Employer offers employees the opportunity to engage fully in challenging work ▪ Employer offers cross-fertilisation of training to meet their career goals as possible. ▪ Employee's career ambition needs to be identified to know the area they have passion for, as this helps to achieve job rotation programs aim. Loyalty increases and they are more engaged because it is associated with their career path (Sanjeev and Surya, 2016)
Protections for wellness, health & safety	It is mandated to protect the safety and health of employees at their workplaces and to promote their physical and mental wellbeing.	▪ Every employer makes sick time provisions. ▪ Where necessary, employers provide access to healthcare in addition to public healthcare support. ▪ Employers limit excessive work requirements and hours of work.
Flexibility, autonomy & control in the workplace	Flexibility is being able to cope and adapt to any change in our surroundings. Flexibility is a must in any compensation plan. Varieties of perceptions which include; perceptions of how they will manage others, expectations of how their employers or managers will manage them, expectations are what each generation brings to the environment of work, and perceptions of how they are to behave.	▪ It should be flexible in a way that allows for change as environmental variables changes from time to time. ▪ When an employee practices flexible working options, he/she feels a sense of responsibility toward whatever has been done around (Salau, 2017).
Equal Employment Opportunities	Fairness means equity. Employees should be entitled to equal and fair treatment when some specific decisions and activities are implemented. Here, organisations are encouraged to develop policies that offer equal pay and equal opportunities.	▪ These include Interactive feedback (communication and exchange of information), clear-cut goals for members of the organisation and periodic equal performance review.
Promotion of employment relationship	Frictions between employer and employee are lessened, and constructive workplace relations are facilitated.	▪ Preserving the relationship is essential. Employment relation promotes an understanding of the problems people have with each other and contributes to greater collaboration between employees and employers.
Respect for culture, Inclusion & Equity	Workforce diversity and the inclusion of less-favoured populations are encouraged, and equity in working conditions is pursued	▪ Organisational culture needs to play a significant role in shaping these attitudes, interests and values (Kagaari, Munene and Ntayi, 2010). ▪ These include: shared values, clarity of vision and mission statement, communication style, etc
Security of employment provisions & predictability	This encourages stable employment prospects for jobs, enables clear communication of employment conditions and ensuring the health of employees is a policy goal	▪ Employer clearly outlines the benefits and risks associated with the conditions of employment. ▪ Employers take open and socially accountable decisions that affect workplace health, such as reduced size. ▪ Workers may gain experience to improve employability

### 2.2 Determinants of employment quality

Armstrong (2010) alluded that the quality of employment has become a salient factor for retaining employees in the workplace. Various inquiries about the dimensions of employment quality include competitive remuneration, equitable rewards, job security, effective communication, role clarity and respect for stipulated terms and conditions of employment (Osibanjo et al., 2016). Yazinski (2019) added favourable learning programme and working atmosphere, work adaptability cost viability, benefits, career advancement, and friendly policies. Most of the perilous issues facing the management of the human resource in the public sector are categorised into five broad areas: work flexibility, employee influence, staff flow, reward systems and work systems. The impact of these practices and strategies when properly adopted helps to improve and boost the organisations. It is against this background that this study has been given an impetus to examine the determinants of employment quality.

#### 2.2.1 Work flexibility

The changing workforce, whose needs and expectations range from Generation X to baby boomers, pose many daunting challenges. Employees’ lifestyle is varied, unique, and continually changing as they try to combine their personal life with their work life. Flexible work arrangements are becoming more and more acceptable. Studies (Osibanjo et al., 2016; Salau et al., 2020) have shown that when an employee enjoys flexible working options, he/she feels a sense of responsibility. The workforce comprises four generations which all possess different viewpoint of work, possession of outstanding ethics of work, and preferred ways of managing and being managed. Flexibility includes programmes such as flexitime, job sharing, and telecommuting. Studies have revealed that workplace flexibility has been hailed as a relatively inexpensive way to aid employees to balance their lives and their livelihoods. It is therefore hypothesised that:

*H*_*1*_*: Work flexibility has a positive effect on employee behavioural outcome*

#### 2.2.2 Organisational culture

Learning an organisation's culture is a vital step during the form of socialisation. An organisation's culture includes values, custom and artefacts a group has in common (Kagaari, Munene and Ntayi, 2010); Samwel, 2018). Culture is also a glue often associated with changing attitude and behaviour of the employees to imitate strategically with evolving culture. With growing difficulty in the attitude of employees, organisations are formulating and implementing policies to achieve individual and organisational goals (Salau et al., 2020; Ibidunni et al., 2016). These policies include career advancement, choice, employment continuity and other opportunities. To achieve dynamic organisation culture, the Nigerian public service needs an enabling and favourable environment. Regrettably, these were not identifiable in the public service of Nigeria. Nevertheless, when the organisational culture is not attractive or unmanageable, this can result in a high turnover of personnel. It is therefore based on this that we have this hypothesis:

*H*_*2*_*: Organisational culture has a positive effect on employee behavioural outcome*

#### 2.2.3 Learning and development

Learning is an integral part of the management of individuals. For continuous improvement, everyone should be provided with opportunities to learn on the job. The main reason for learning and development is for increasing employee's knowledge (Ibidunni et al., 2016). We learn every day, so learning is an activity that needs to be done continuously to avoid employees having obsolete information or change idea. (Falola, Olokundun, Salau, Oludayo and Ibidunni 2018) thinks it is any activity aimed at advancing employees abilities and competence (Pearce, 2010). All programmes for learning has to be based on need and well-tailored towards achieving organisational objectives and goals. If learning and development are correctly done, it benefits to be of great advantage to organisations and individuals in the long run. A developmental programme helps to improve specific abilities that every employee needs to elevate (Armstrong, 2010). Training aids an employee in terms of increasing knowledge and understanding of how the business operates and what it takes to succeed no matter the competition. To increase employee and organisation reciprocity, it is necessary to structure learning adequately (Nahavandí, Denhardt, Denhardt and Aristigueta, 2015; CIPD, 2007). The importance of learning from work-related problems, especially in public services, is permissibly undermined and isolated from emerging trends. It can therefore be hypothesised that:

*H*_*3*_*: Learning and development have a positive effect on employee behavioural outcome*

#### 2.2.4 Involvement and rewards

Employee involvement has to do with the effective participation of employees in their job to use their skills to achieve increased performance and high organisational productivity. It also refers to an employee's active contribution in making decisions and solving organisational problems and job autonomy. Due to this, it is expected that employees are loyal and committed, satisfied and ensure high productivity (Onukwube et al., 2010; Ahmed et al., 2011; Govaerts et al., 2011). Ensuring employee's involvement is impossible in an organisation that uses a mechanistic approach. The motivation of the employees requires a flexible reward strategy that reacts in conjunction with the competitive rewards (Bloom and Van Reenen, 2011; Armstrong, 2001) to issues that are both in-house (such as the qualifications of the competent staff, resources and allocation) and outside (such as government minimum wage policies). The reward could come as a financial or a non-financial form of compensation given to employees or workers for services offered (Osibanjo et al., 2014). According to Akpa, Egwakhe and Aliu (2020), a proper compensation/reward system is made up of base compensation, reward incentives and indirect compensation. The presence of a transparent and structured reward system may encourage, attract and retain employees most especially gifted talents in an organisation and would encourage an increase in organisational productivity (Salau et al., 2020). It constitutes wages/salaries, incentives, fringe benefits, bonuses etc. When employees are well compensated/rewarded employees, exceptionally gifted employees are retained in an organisation. It is only when employees are adequately satisfied with their organisation that they would stay within the work environment for a broader length of time (Shakeel and But, 2015; Akpa, Egwakhe and Aliu, 2020). Based on this, it is hypothesised that:

*H*_*4*_*: Involvement and rewards have a positive effect on employee behavioural outcome*

#### 2.2.5 Work systems

The nature of the work system has made tasks more intrinsically exciting and rewarding by providing autonomy, flexibility, diversity of skill, job identity, feedback, personal growth and fulfilment. Where the functional work system is non-existent in an organisation makes it difficult to monitor the activities of the organisation by external shareholders (Akpa, Egwakhe and Aliu, 2020). An understanding of how job contents can make a significant contribution to employee satisfaction and commitment is a significant issue of concern for the management of Nigerian public service. Based on the above, the hypothesis for this study is that:

*H*_*5*_*: Work system has a positive effect on employee behavioural outcome*

#### 2.2.6 Work relationship

Relationships are a vital element in the organisational life structure. Work is an inherently relativistic act with relationships that play an essential part in developing a viable and meaningful work life. Decisions and experiences and reactions to the working world are understood, affected and influenced both within and outside the workplace by relations with others. Preserving relationship is crucial as it helps to promote an understanding of the problems people have with each other and contributes to greater collaboration between employers and employees. Workers from particular social links with their supervisors, colleagues, customers, team members and people outside the workplace. Each of these relationships inhabits a specific niche in the lives of employees. It can therefore be hypothesised that:

*H*_*6*_*: Work relationship has a positive effect on employee behavioural outcome*

### 2.3 Employee behavioural outcomes

Employee behavioural outcomes is a central issue of the internal and cross-border business environment of this modern era. Across the business world, there are consistent and increasing changes (such as technology, structure, process and policies) and a competition faced by companies in different industries. Among companies, globalisation poses unique opportunities and challenges in different industries and so for these companies to benefit and survive, they employ capable hands that would produce the required positive behavioural outcomes (Sanjeev and Surya, 2016; Waribo et al., 2019). Employee behavioural Outcomes, in this context, refers to as the various sequences of actions (positive or negative) carried out by a company's workforce to performing a task having an outcome that has the potential to affect the organisation. The concept of employee behavioural outcomes to many organisations is like food to human life. Many organisations around the world rely on the abilities of human resources to reach enhanced organisational performance and competitive advantages through controlled behavioural outcomes employees express at work. Notably, organisations in competitive situations are in constant need of a workforce who can positively react to bring about healthy organisational performance (Bireswari, 2013; Samwel, 2018).

Current winners in the global market scene are organisations that have been transformed by their employees through positive behavioural outcomes employees exhibit in the workplace (Osibanjo et al., 2014; Ismail et al., 2018). These behavioural outcomes include a high level of engagement, loyalty, satisfaction, intention to stay, and performance among others (Sanjeev and Surya, 2016; Osibanjo et al., 2018; Samwel, 2018). Emphasised also, are current business organisations that are presently focusing on factors that provide a sustainable competitive edge in the shape of positive behavioural employees’ outcomes of which are expressed by a highly empowered employee (Slamet, 2019). That is to say; positive behavioural outcomes are acceptable in the workplace (Waribo et al., 2019).

Positive employee behavioural outcomes are in the form of low intention to quit, high job satisfaction and loyalty among others (Osibanjo et al., 2018; Falola, Olokundun, Salau, Oludayo and Ibidunni, 2018). Adding to this, several forces drive these behavioural reactions of employees either from the external and internal business environment or both as they attempt to carry out their workplace duties (Waribo et al., 2019). Consequently, employee behavioural outcomes is often a function of situational experience and the individual in question (Yuvaraj and Nadheya, 2018). Precisely, employee displays several kinds of reactions when it comes to change such as cognitive, intentional, affective and behavioural. In other words, positive human resource behavioural outcomes seek to enhance the people-organisation relationship and reach organisational goals (Joo and Park, 2010; Shakeel and But, 2015; Olawale and Olanrewaju, 2016).

In relation, the behaviours people show at work are diverse and are nearly unlimited. For instance, they celebrate successes together, conspire against their superiors, tell various stories at work, comfort each other in the case of failure, avoid work, develop peculiar rituals or knowingly exhibit behaviour patterns that shake existing norms which may form behavioural outcomes that are for or against the interest of the organisation (Islam and Rahman, 2016; Samwel, 2018). In another study, (Dan-Jumbo 2018; Ali, Kakakhel, Rahman and Ahsan 2014) opined that the behavioural outcomes of employees (employees’ satisfaction, turnover intention, commitment, citizenship behaviour and perceived performance) have very significant bearings on the organisational operation notwithstanding its nature and size. Supporting this, Olori and Dan-Jumbo (2017), asserted that both public and private organisation seek the daily involvement of employees with more significant positive job behavioural outcomes such as satisfaction, identification, commitment, loyalty and lower adverse work outcomes such as quitting intention, incivility and dishonesty (Triana, Jayasinghe and Pieper, 2015; Sanjeev and Surya, 2016).

### 2.4 Nigerian Public service defined

The Nigerian Public Service originates from organisations established by the British in colonial times. Public service meaning is written down in Section 277 (91) of the Constitution of the Federal Republic of Nigeria of 1979, as it encompasses the civil service (ministries), statutory or para-state bodies, the judiciary, the legislature, the educational institutions, the police, the teaching services and the various financial institutions in whole or in part. Public service roles include: (1) implementation and execution of government socio-economic and political policies; (2) structuring, planning and executing public service; (3) increasing government revenues to carry out its activities; (4) maintaining administrative, political and financial accountability; and (5) monitoring and reviewing organisational performance. Nigeria's public service has come across many problems. The problems hamper public service efficiency and productivity. The Nigerian public service has an endemic disease, however. There was a spirit of camaraderie, neutrality, efficiency and so on before military intervention in politics in 1966. Strong and well-healed governments were the enthusiasm and the dynamism that followed the Nigerian public service, which was until now one of the leading public services. So Nigeria's political climate became unfortunate with the emergence of the military in politics. These are some of the challenges facing the profession, and one of the quickest ways to improve them is to strengthen the institution of public service as they are the leading employers of administrators. Without independence and adequate funding, the implementation of government policies can be manipulated and done hazardously, leading to poor policy execution and lack of loyalty.

## 3 Methodology

The adopted descriptive design made use of survey method based on the impracticability of studying the entire large population of public (corporation) service in Nigeria. In essence, the survey method enabled the researcher to make inferences that apply to the entire population, based on Cooper and Schindler (2003) assertion that survey research is an appropriate method to generalise from a sample to a population. Krejcie and Morgan (1970). Sample Size Determinant Table was used to determine the sample size. The total study population is 3,727, as shown in Table 3.2 above. On the Krejcie and Morgan Table, the population of 3,727 at 95% confidence level falls within 5th row/9th column. Therefore 350 as recommended on the table was achieved. Thus, to ensure adequate sample size representation, the total sample size for the study was 350.

A descriptive system of study has been used in this review because it helps to achieve a reasonable representation of the various sub-groups in the population. The selection of the agencies involved was based on their performance. The stratified random method was adopted to help to increase the efficacy of samples. As a result, the population is categorised into various strata (selection of staff across departments/levels). The survey was performed using a questionnaire. The questionnaire items were modified and adopted standard questions based on items from the validated survey works of previous scholars in similar subject areas.

The questionnaire adopted a five-scale Likert format to capture the exact level of consideration and responses to the probing item. Represented thus: 1 = Strongly Disagree (SD); 2 = Disagree (D); 3 = Undecided (U); 4 = Agree (A); and 5 = Strongly Agree (SA). The questionnaire consisted of eight sections: A, B, C, D, E, F, G and H. Section ‘A’ consists of the demographic profile of the respondents, Section ‘B’ includes questions about work flexibility, and Section ‘C’ focuses on corporate culture, and Section ‘D’ contains questions about learning and development and Section ‘E’ captures items on involvement and rewards; section ‘F’ emphasises on work systems; Section ‘G’ looked at work relationship and finally, Section ‘H’ concentrates on the dependent variable (employee behavioural outcomes).

The use of this scale in quantitative research enabled numerical representation and management of observations to clarify and relate the mindsets signified by the observations. By standard, the Likert scale posits that the weight accorded experience by anybody is linear and is graduated from strongly agree to strongly disagree with the additional postulation that attitudes are measurable (Samwel, 2018). Another fundamental importance of Likert scale adoption in the realisation of the objective of this study is that the assignment of the numeric value to qualitative responses from questionnaire allows subjection of the responses to descriptive and inferential statistics.

The principal investigator submitted the survey questionnaire to the Business Management Research Ethics Committee for ethical approval. This was approved on December 10, 2019, with approval number by BMREC 19/28/303). A letter of introduction was given to the research team, which was presented to selected government-owned parastatals stating the purpose of the research. The significance of this study was adequately indicated. Our paper complied with the ethical principles as stipulated by the Covenant University Business Management ethics committee requirements in the process of data collection and their analysis. Of importance is that the authors made it a point of duty to guarantee that the data gathered were treated as anonymous and confidential. The participants in the study were all well informed of their free choice to partake or refuse; hence this gave them more confidence to express their consent. Ethical issues such as the right of respondents to privacy and free-will were envisaged while the potential risks of possible physical harm and unanticipated measures were provided for.

It is equally imperative to note that verbal consent was gotten from the selected respondents (staff of the selected public sector in Nigeria) of this research. The establishment departments of the selected government-owned parastatals/corporations were consulted for research permission guidelines. Based on the information provided in principle, an application letter was written requesting permission to research their institutions with the objective of the study clearly stated. Also, the research ethics approval form was attached to the application letter. This type of research was categorised as **exempt research** that involves a survey with no or minimal risk, i.e. level 1 research as presented in the Research Ethical Application Form. In the spirit of anonymity and confidentiality, exempt research work in management sciences does not require signed consent from the participants but implied consent is usually enough. By verbal consent, the researchers ensured that the respondents were well informed about the context and purpose of this research, and kept abreast of the participation process.

**Figure 1:**
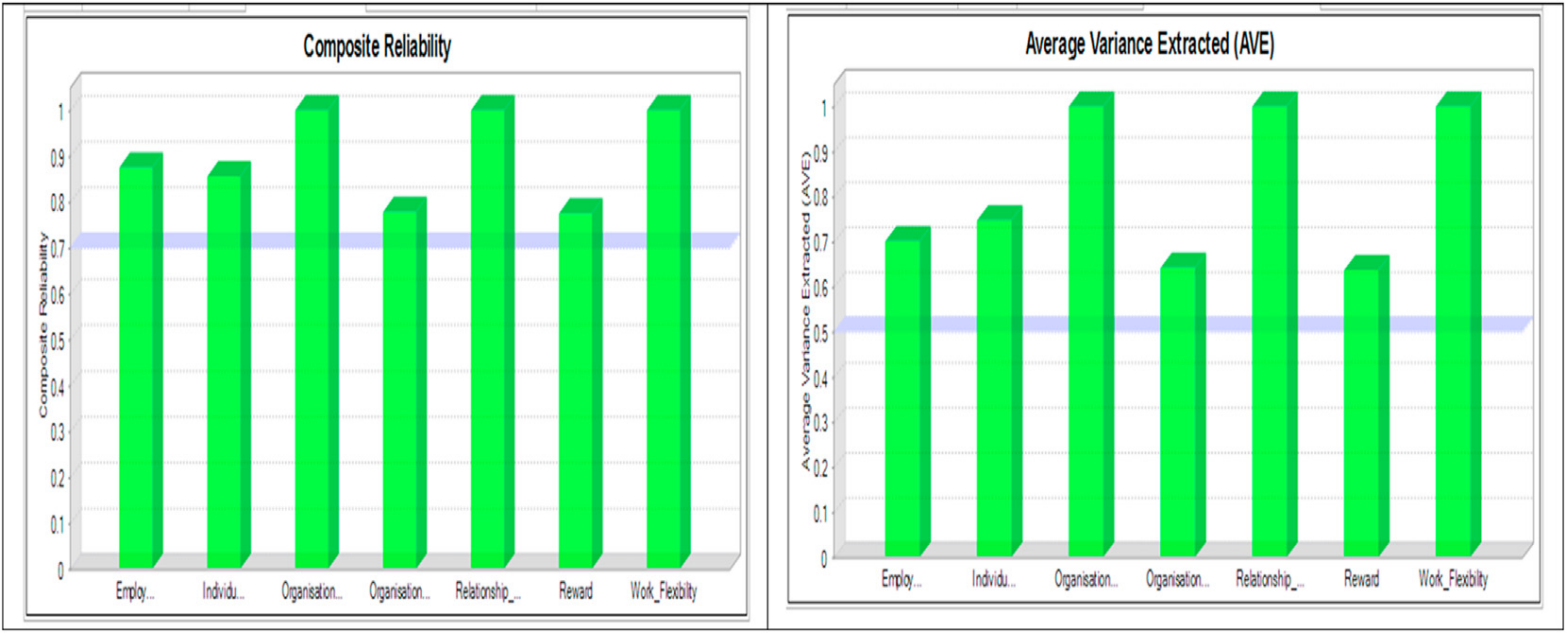
Composite reliability and average variance extracted.

To test the internal consistency and homogeneity of the items, Cronbach's Alpha, composite reliability and average variance extracted coefficients (AVE) were used (Hair, Anderson, Tatham and Black, 2006). In the overall, the Mean Cronbach's alpha of all constructs measuring corporate reputation and organisational competitiveness affirmed that the constructs were reliable. This was so having scaled the set minimum value of 0.75 that was necessary to indicate that the instrument was both internally consistent and reliable. The composite reliability and average variance extracted was computed for this study as recommended by Fornell and Larker, composite reliability acceptable when the values are above 0.7. In contrast, the average variance extracted satisfactorily when the values are above 0.5. The results are presented in Figure 1.

## 4 Results and discussion

The main drive of this research was to explore the effects and relationship between employment quality and behavioural outcomes in selected public service. Meanwhile, 350 copies of the questionnaire were given out to the selected agencies out of which 230 copies of the questionnaire, representing 66%, were retrieved and adjudged suitable for the analysis. The response rate is demonstrated in Table 2, and this was done based on the type of Public Service Corporations in Nigeria.

**Table 2:** Respondents by type of public service corporations in Nigeria.

Region	Agencies of Federal government	Questionnaire Distributed	Questionnaire Retrieved for Analysis	Total % of questionnaire Analysed
**Maritime**	Nigerian Ports uthority (NPA)	193	126	65.3%
**Health**	National Agency for Food and Drug Administration and Control (NAFDAC)	157	104	66.2%
**Overall Total**		**350**	**230**	**100.0%**
% of Response rate			**66%**	

Inferences from Table 3 indicated that 62.5% of the respondents were male, while 37.5% were female. The purpose of including the gender of respondents was to determine whether there is gender balance in the employment quality of public service. The table also shows that 64.7% of the respondents are within the age bracket 20–39. Furthermore, Table 3 shows that 65.9% of respondents are married, while 33.5% are single. On educational qualification, it was indicated that all the respondents are educated and this, in turn, means that they are qualified enough to provide reasonable answers to the items in the questionnaire. Finally, for the work experience, this study indicated that 31.2% of the respondents had spent less than six years in the public service; while 45.3% claimed 6–10 years and only 14.7% of the respondents have spent beyond ten years in the public service.

**Table 3:** The demographic composition of the sample N = 230.

Characteristics	Sub-Profile	Percentage
Gender	Male	62.5
	Female	37.5
Age	20–29 years	28.8
	30–39 years	35.9
	40–49 years	24.7
	50 years and above	10.6
Marital Status	Married	65.9
	Single	33.5
	Others	0.6
Educational Qualification	NCE/OND	29.4
	Bachelors' Degree	55.7
	Masters' Degree	12.0
	Doctorate Degree	2.9
Work Experience	0–5 years	31.2
	6–10 years	45.3
	11–15 years	14.7
	Above 15 years	8.8

*Note:* Total number of respondent = 230. A first-order measurement model was conducted to analyse the covariance between constructs, items loadings and goodness of fit index. The initial indicated statistically inadequate goodness-of-fit index (χ2/df = 2.231, CFI = .967, TLI = 941, RMSEA = .010) as presented in Table 4. The measurement model proved to be correct in terms of the covariance matrix, as the factor loadings are substantial statistically. Thus; the model was free from offending error.

Moreover, the Cronbach's alpha of the first-order factors were .852 (employment quality) and .824 (employee work behaviour). The data supported the model adequacy in terms of their convergent and discriminant validity. Second-order estimation of the six constructs that measured employment quality indicated a statistically significant goodness-of-fit, as demonstrated in Table 4. The model fit indices satisfied their critical threshold, which indicated a fitting relationship management model. Table 5 demonstrated the model fit summary.

**Table 4:** The model fit summary.

Model-Fit Index	Score	Cut-off Values
Chi-square/Degree of Freedom (CMIN/DF)	2.321	2, 3, or 5 upper limit
Normed Fit Index (NFI)	.953	=/> .90
Comparative Fit Index (CFI)	.967	=/> .90
Root Mean Squared Error of Approximation (RMSEA)	.010	.05 or less = good
Goodness of Fit (GFI)	.964	= > .90

**Table 5:** Regression weights of the sample.

Dependent var.		Independent indicators	Estimate	S.E.	C.R.	P	Hypotheses
Behavioural Outcomes	≤	Work flexibility	.222	.068	3.279	.001	H1 – Accept
Behavioural Outcomes	≤	Organisational policy	.125	.097	2.167	.030	H5 – Accept
Behavioural Outcomes	≤	Learning & development	.370	.011	4.569	.000	H3 – Accept
Behavioural Outcomes	≤	Organisational culture	.138	.055	2.062	.006	H2 – Accept
Behavioural Outcomes	≤	Equitable rewards	.161	.081	2.099	.005	H4 – Accept
Behavioural Outcomes	≤	Relationship with others	.168	.027	2.335	.044	H6 – Accept

**Figure 2:**
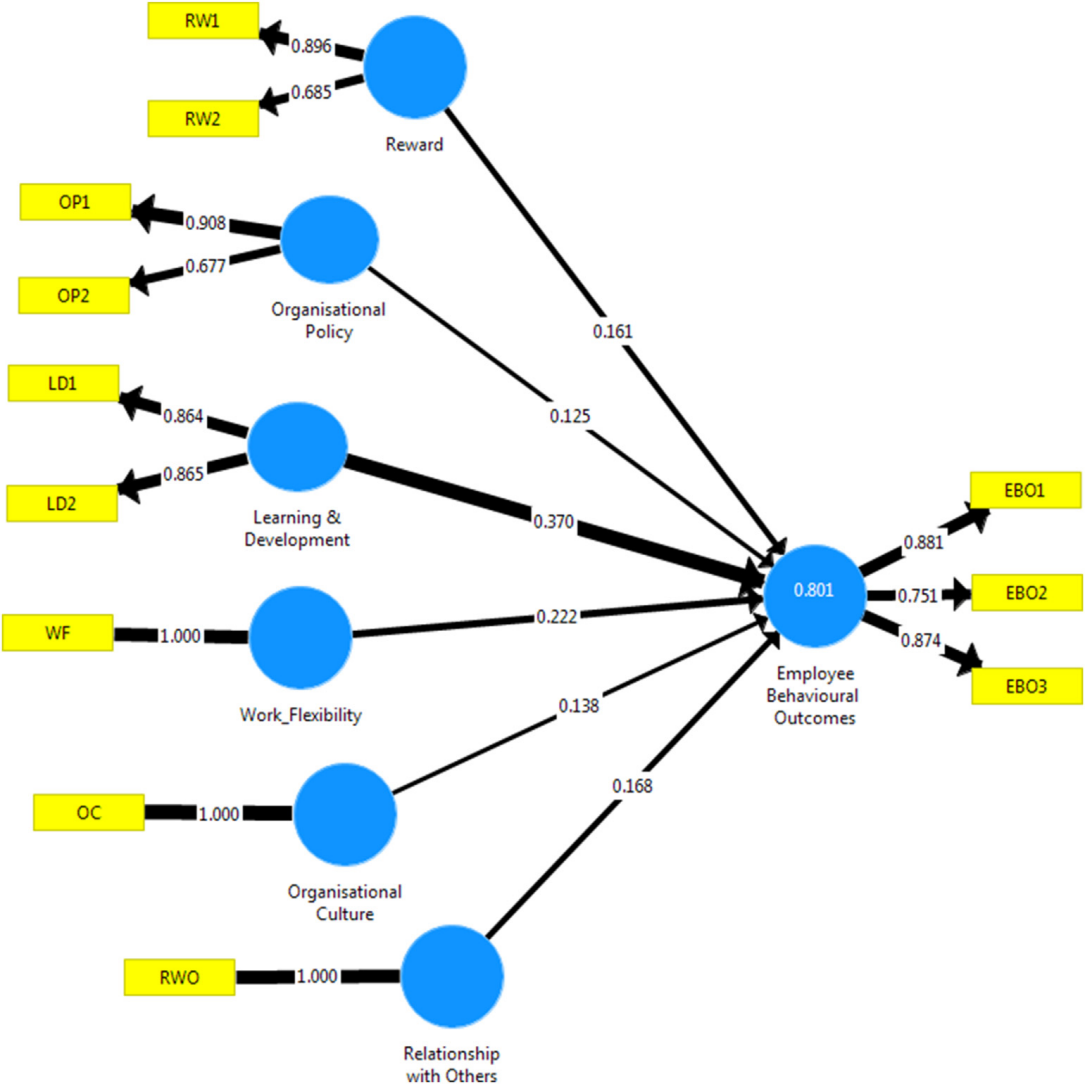
Standardised algorithm estimates of structural model.

**Figure 3:**
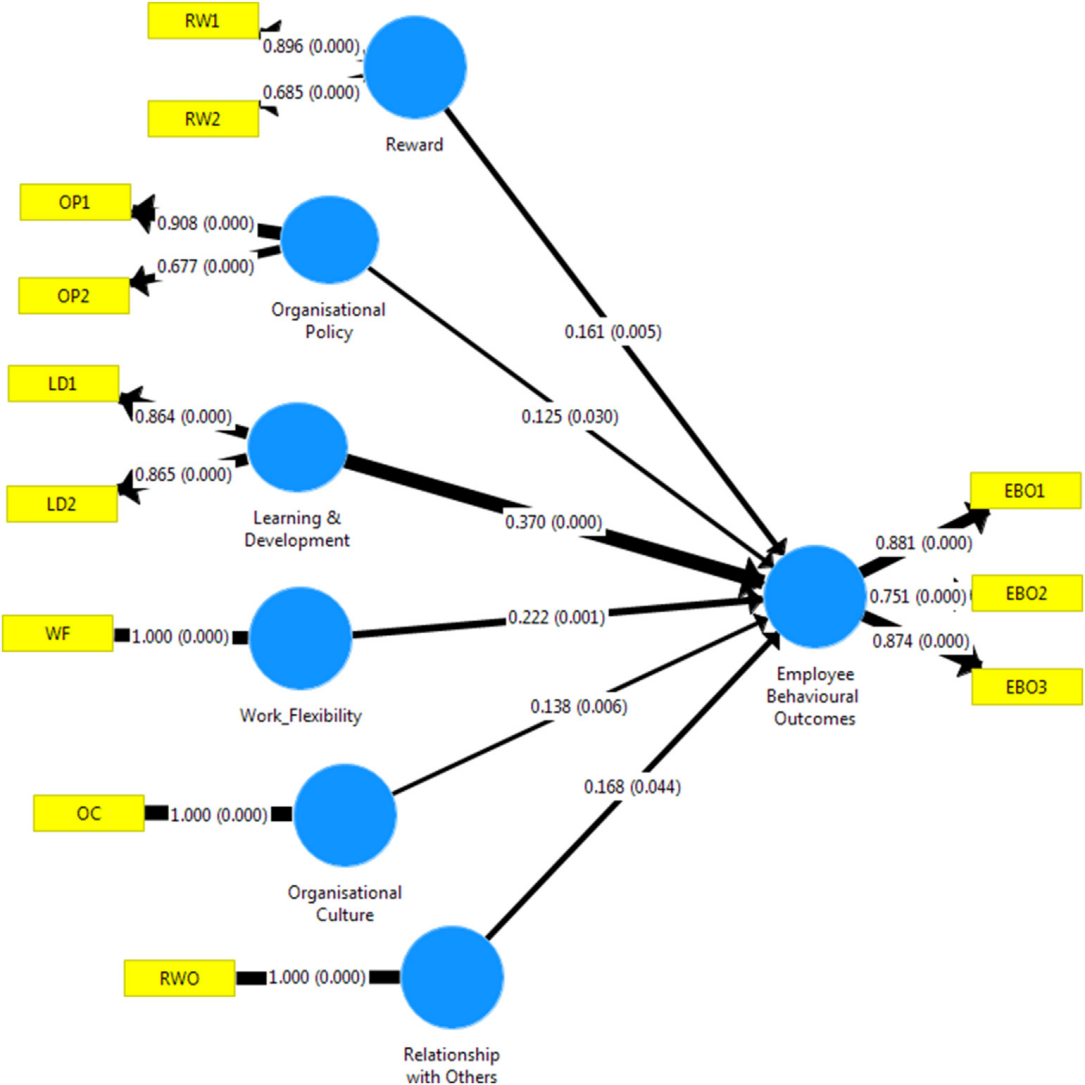
Standardized P-value estimates of structural model.

The goodness of fit helps to explain the closeness that exists between the observed variables and expected values (Bentler and Liang, 2008). To complement the goodness of fit model, standardised estimates of the structural model are presented in Figures 2 and 3, respectively.

Figures 2 and 3 depict standardised P-value estimates of Structural Model in the study. Based on the coefficient scores, it is evident that there is a close association that exists amongst the variables (work flexibility, organisational policy, learning and development, organisational culture, reward system, and work relationship). In other words, it is crucial to note that the set of observed variables such as learning and development, work relationships, reward system, organisational culture and policy, and work flexibility have a positive association with employee behavioural outcome. In Figure 2, the parameter estimate indicates that learning and development (.370, p < 0.05) and work flexibility (.222, p < 0.05) are the most significant predictor of employee behavioural outcomes. On the other hand, an organisational policy is expected to have a strong positive relationship with employee behavioural outcome. Still, based on the result, organisational policies have the least predictive estimates of employee behavioural outcome, which suggests that the more rigid the organisational policy is encouraged. This lesser positive behavioural outcome will be derived from the job. Hence, this finding contradicts the study of Armstrong (2001), who claimed that organisational policy goes a long way in stimulating employees’ attitude and enriching jobs towards more incredible performance.

Work flexibility (H_1a_), learning and development (H_2a_), organisational culture (H_1b_), involvement in the determination of reward system (H_3a_), and work relationship (H_3b_) were found to be directly imported in the prediction of employee behavioural outcomes. Therefore, the hypothesised statements are accepted. Organisational policy (H_2b_) had the least value (0.125), though the study established a positive and significant influence on employee behavioural outcome. Kruskal Wallis Test was also carried out to depict the difference in employees’ perception of employee behavioural outcome as demonstrated in Table 6.

**Table 6:** Variation in employee behavioural outcome across respondents’ demographic characteristics.

	Gender	Age	Educational Qualification	Marital status	Experience
Chi-Square	7.808	5.741	8.723	6.731	9.672
Df	4	4	4	4	4
Asymp. Sig.	.099	.020	.018	.033	.039

Using employee behavioural outcome (dependent variable) and demographical characteristics of respondents (independent variable) which include gender, age, marital status, educational background, and experience of employees) as the independent variables. Kruskal Wallis Test was carried out, which showed the difference in employees' perception of employment quality on employee behavioural outcome by gender (λ^2^ = 7.808, df = 4, P > 0.05) is not statistically significant. The result portray that the difference in employees' perception of employment quality on employee behavioural outcome by age (λ^2^ = 5.741, df = 4, P < 0.05), educational background (λ^2^ = 7.723, df = 4, P < 0.05), marital status (λ^2^ = 6.731, df = 4, P < 0.05), and work experience (λ^2^ = 9.672, df = 4, P < 0.05) are statistically substantial. This implies that the differences in employees’ perception can be linked to age, educational background and work experience. With specific reference to experience and level of educational background.

## 5 Discussion

The discussions on the research questions were also presented below:

i. *Research 1: To what extent can work flexibility and organisational culture influence employee behavioural outcomes?* The result of the test indicated that work flexibility (β = .222, p = .001) and organisational culture (β = .138, p = .006) have a moderate and positive impact on employee behavioural outcomes at 95% confidence level. This implies that a unit change in work flexibility and organisational culture will undoubtedly lead to an increase in employee behavioural outcomes by 22.2% and 13.8% respectively. This finding that showed significant and direct relationship among work flexibility, organisational culture and employee behavioural outcomes are consistent with previous studies (Akinyele, 2010; Awan and Mahmood, 2010; Chandrasekar, 2011; Bireswari, 2013).
ii. *Research 2: In what ways can learning and development and organisational policy affect employee behavioural outcomes?* The results suggested that learning and development (β = .370, p = .000) and organisational policy (β = .125, p = .030) positively and significantly affect employee behavioural outcomes at 95% confidence level. This predicted that a unit change in learning and development and organisational policy would positively lead to an increase in employee behavioural outcomes by 37% and 12.5% respectively. This result extended the works of Joo and Park (2010) and Leblebici (2012) who found strong evidence in support of a significant association capacity development, work systems and employee behavioural outcomes. This was also evident in the works of Fatimah et al. (2012), and Sanjeev and Surya (2016).
iii. *Research 3: To what extent can equitable rewards and work relationship influence employee behavioural outcomes?* The results suggested that equitable rewards (β = .161, p = .005) and work relationship (β = .168, p = .044) positively and significantly affects employee behavioural outcomes at 95% confidence level. This predicted that a unit change in equitable rewards and work relationship would positively lead to an increase in employee behavioural outcomes by 16.1% and 16.8% respectively. The present outcome is in line with the study by Anvari et al. (2011) who investigated the relationship between strategic compensation practices and affective organisational commitment. This relationship finds strong support in extant literature (Depedri et al., 2010; Galanou et al., 2011; Olawale and Olanrewaju, 2016; Ismail et al., 2018).
iv. *Research 4: What are the employees' perceptions of employment quality and employee behavioural outcomes across the demographic characteristics?* Kruskal Wallis Test was carried out to show the variation in employee behavioural outcome across respondents' demographic characteristics. The result shows a significant difference in the perception of employee behavioural outcome by age, educational background, marital status, and work experience. While perception of employee behavioural outcome by gender is not statistically significant. The current study finding on the variations in the perception of behavioural outcome across demographic characteristics was similar to previous findings (Adeniji and Osibanjo, 2012; Abdulla et al., 2010).

Extant literature has shown that organisational policies play a vital role in employees’ satisfaction. The results from this survey are contrary to past studies in which strong significant relationships were acknowledged between organisational policies and performance (Sanjeev and Surya, 2016; Fatimah et al., 2012). This is also consistent with the Fatimah et al. (2012) and (Samwel (2018) findings that capacity development and equitable rewards should be seen as a strategic tool for competitive positioning. Therefore allowing the worker the opportunity to learn more about their necessary and planned role would improve the performance of the individual worker and allow them to feel that they are part of the organisation. The importance of providing workers with equitable rewards and harmonious relationship can never be overlooked, hence the need for organisations to ensure that the indicators of employment quality as identified in this study are focused on, emphasised and given adequate attention.

## 6 Managerial implications and conclusion

The primary objective of this study is to identify the relationship between employment quality and job satisfaction using the Nigerian public sector. It is evident that employment quality influences employee satisfaction. Thus, the study provides more understandings on the effect of employment quality, taking into consideration variables like work flexibility, learning and development, organisational culture, organisational policy, involvement in the determination of reward system, and work relationship on employee satisfaction. By implication, employment quality influence satisfaction within organisations. Therefore, managers must pay extra attention to work flexibility, learning and development, organisational culture, involvement in the determination of the reward system, and work relationship. Although based on the findings, it was discovered that organisational policy has a significant inverse effect on employee behavioural outcomes which serves as an advantage to increase employees’ efficiency.

Further, it can be inferred from the findings that an organisation cannot prosper well, thrive, expand or even survive without addressing the issue of poor attitude appropriately. The adverse behavioural outcomes experienced in the public service will be history if employees are well educated, qualified, and confident about their job security, promoted, and given a sense of value by management. This paper offers practical implication and concludes that the deficiencies witnessed in Nigeria's public sector are attributed to issues such as corruption, bureaucratic administrative machinery, policy reversals, the insufficient capacity of the majority of civil servants, certificate forgery to get promotion and gain entry, age falsification to remain in service beyond the stipulated age, primordial considerations like ethnicity, etc. By implications, this study suggests that organisational policies should be tailored towards ensuring employment quality that will facilitate commitment and positive work behaviour.

## Declarations

### Author contribution statement

O. Salau: Conceived and designed the experiments; Analyzed and interpreted the data.

A. Osibanjo: Contributed reagents, materials, analysis tools or data; Wrote the paper.

A. Adeniji: Conceived and designed the experiments; Analyzed and interpreted the data; Contributed reagents, materials, analysis tools or data; Wrote the paper.

H. Falola: Performed the experiments; Contributed reagents, materials, analysis tools or data; Wrote the paper.

E. Igbinoba, T. Atolagbe and O. Ogueyungbo: Analyzed and interpreted the data; Contributed reagents, materials, analysis tools or data; Wrote the paper.

### Funding statement

This research did not receive any specific grant from funding agencies in the public, commercial, or not-for-profit sectors.

### Declaration of interests statement

The authors declare no conflict of interest.

### Additional information

No additional information is available for this paper.
